# Lipoprotein(a) and Prognostic Nutritional Index Levels Exhibit Interactive and Joint Effects on Mortality Risk in Patients With Coronary Artery Disease: Findings From a Real-World Study

**DOI:** 10.31083/RCM51948

**Published:** 2026-10-16

**Authors:** Jinming Chen, Xiaozhao Lu, Xianlin Ruan, Zeliang Li, Yimidiguli Aji, Ahmed Abdulwasea Furas Ali Mohsen, Wei Guo, Jiyan Chen, Shiqun Chen, Junyan Xu, Jin Liu, Yong Liu

**Affiliations:** ^1^Department of Cardiology, Guangdong Provincial People’s Hospital (Guangdong Academy of Medical Sciences), Southern Medical University, 510080 Guangzhou, Guangdong, China; ^2^Guangdong Provincial Key Laboratory of Coronary Heart Disease Prevention, Guangdong Cardiovascular Institute, Guangdong Provincial People’s Hospital, Guangdong Academy of Medical Sciences, 510080 Guangzhou, Guangdong, China; ^3^Department of Cardiac Intensive Care Unit, The First People's Hospital of Kashi Prefecture, 844000 Kashi, Xinjiang, China; ^4^Guangdong Provincial Geriatrics Institute, Guangdong Provincial People’s Hospital (Guangdong Academy of Medical Sciences), Southern Medical University, 510080 Guangzhou, Guangdong, China; ^5^Global Health Research Center, Guangdong Provincial People’s Hospital, Guangdong Academy of Medical Science, Southern Medical University, 510080 Guangzhou, Guangdong, China

**Keywords:** coronary artery disease, lipoprotein(a), prognostic nutritional index, additive interaction, all-cause mortality, cardiovascular mortality, risk stratification, real-world study

## Abstract

**Background:**

Abnormal lipoprotein(a) (Lp(a)) levels and malnutrition are independent cardiovascular risk factors; however, the associated combined effects on mortality in patients with coronary artery disease (CAD) remain incompletely understood. Thus, this study aimed to evaluate the independent and joint associations of Lp(a) and the prognostic nutritional index (PNI) with all-cause and cardiovascular (CV) mortality in patients with CAD, as well as the associated combined interaction.

**Methods:**

A total of 47,482 patients with CAD from the Southern China multicenter Cardiorenal Improvement II cohort (2007–2020) were enrolled. Patients were stratified according to Lp(a) levels (low: <15 mg/dL; middle: 15 to <30 mg/dL; high: ≥30 mg/dL) and PNI (high: ≥45; low: <45). The primary outcomes were all-cause mortality and CV mortality. Restricted cubic splines were used to assess non-linear associations, while multivariable Cox models and Kaplan–Meier analyses were used for risk assessment; receiver operating characteristic (ROC) curves were used to compare predictive performance, and additive interaction analysis was used to evaluate joint effects.

**Results:**

During a median follow-up of 5.2 years, 5229 patients (11.01%) died from any cause and 3698 died from CV causes. In fully adjusted models, high Lp(a) combined with low PNI was associated with the highest risk of all-cause mortality (hazard ratio (HR) 1.85, 95% confidence interval (CI) 1.69–2.03) and CV mortality (HR 2.20, 95% CI 1.98–2.46), compared with the low Lp(a)/high PNI reference group. The combined model achieved a higher area under the receiver operating characteristic (AUROC) curve for mortality prediction than either marker alone (*p *< 0.05). Significant additive interactions between Lp(a) and PNI were observed for all-cause mortality (relative excess risk due to interaction, RERI 0.30, 95% CI 0.14–0.43) and CV mortality (RERI 0.32, 95% CI 0.11–0.52).

**Conclusions:**

Lp(a) and PNI are independently associated with increased long-term mortality in patients with CAD and demonstrate a significant additive interaction. Joint assessment of Lp(a) and PNI may help identify high-risk patients with CAD at hospital discharge and may inform clinical risk stratification, optimized management, and personalized follow-up.

**Clinical Trial Registration:**

NCT05050877, https://clinicaltrials.gov/study/NCT05050877?cond=NCT05050877&viewType=Card&rank=1.

## 1. Introduction

Despite widespread application of percutaneous coronary intervention (PCI) and guideline-directed lipid-lowering therapy, coronary artery disease (CAD) is a major global public burden challenge [1,2]. Traditional low-density lipoprotein cholesterol (LDL-C)-centered management cannot fully eliminate residual cardiovascular risk, and factors beyond conventional lipid targets play critical roles in CAD progression and prognosis [3]. Lipoprotein(a) [Lp(a)] has emerged as an independent, causal risk factor for atherosclerotic cardiovascular disease and as a powerful prognostic marker in CAD [4]. Clinical trials and population studies have demonstrated that elevated Lp(a) is associated with an increased risk of adverse cardiovascular event risk even with well-controlled LDL-C levels [5,6,7].

In addition to lipid metabolic abnormalities, malnutrition independently predicts worse long-term cardiovascular outcomes [8]. The prognostic nutritional index (PNI), a composite indicator calculated from serum albumin concentration and peripheral lymphocyte count, is one of the most widely used nutritional assessment tools in clinical research [9,10,11]. Compared with other nutritional screening tools, PNI has the advantages of being objective, simple to calculate, and readily available from routine blood tests, making it particularly suitable for large-scale epidemiological studies. Accumulating studies have validated the prognostic value of PNI in various atherosclerotic cardiovascular diseases, including CAD, heart failure and aortic dissection.

Both Lp(a) and PNI are well-recognized independent prognostic biomarkers for CAD, whereas their combined predictive effect on long-term CAD prognosis remains unclear. It is well established that Lp(a) exerts pro-atherogenic effects by delivering oxidized phospholipids that provoke vascular inflammation and oxidative damage [12]. As a marker of nutritional and immune status, PNI can reflect patients’ overall physical conditions to a certain extent [13,14,15]. Malnutrition can increase systemic inflammation, which may further exacerbate vascular injury mediated by Lp(a) [16,17]. These observation suggest a potential pathological interaction between Lp(a) and PNI.

However, most previous studies have only investigated the independent prognostic effects of Lp(a) or PNI in CAD patients separately. The combined effects and potential interaction between Lp(a) and PNI on CAD mortality have not been systematically investigated to date, thus representing a critical research gap. Therefore, the aim of this study was to determine the independent associations of Lp(a) and PNI with all-cause and cardiovascular mortality in CAD patients, and to explore their joint effects on mortality risks. This research was based on a large-scale, multicenter real-world cohort of CAD patients in Southern China. Our findings may offer new perspectives and scientific evidence for developing nutritional interventions and for optimizing prognostic strategies in CAD.

## 2. Methods

### 2.1 Study Design and Study Population

This study utilized data from the CardioRenal Improvement II (CIN-II) cohort, a large-scale, multicenter retrospective observational cohort study conducted at 5 major tertiary care centers in Southern China, with consecutive patient enrollment between January 2007 and December 2020 (ClinicalTrials.gov NCT05050877, https://clinicaltrials.gov/study/NCT05050877?cond=NCT05050877&viewType=Card&rank=1). We initially screened 145,267 patients who underwent coronary angiography (CAG) or percutaneous coronary intervention (PCI). Of these screened individuals, 99,699 patients received a definitive diagnosis of CAD, as confirmed by CAG. Diagnostic criteria were defined as ≥50% diameter stenosis in any major epicardial coronary artery, including the left main, left anterior descending, left circumflex, and right coronary arteries. Indications for CAG and PCI procedures were determined by symptoms of ischemia, elevated cardiac markers, or electrocardiographic changes, with all procedures performed in accordance with standardized clinical practice guidelines [18,19]. Coronary angiograms were interpreted by experienced cardiologists following unified diagnostic specifications, ensuring consistent diagnostic standards across multiple centers. Patients were excluded if they met any of the following criteria: (1) aged <18 years (n = 638); (2) incomplete or missing follow-up data (n = 5759); (3) diagnosis of severe sepsis (n = 80) or malignant tumor (n = 1382); (4) lack of data on plasma Lp(a), serum albumin, absolute lymphocyte counts or PNI (n = 44,358). A total of 47,482 patients with CAD were included in the final analysis. The study design and screening process are presented in Fig. 1. This study was approved by the Ethics Committee of Guangdong Provincial People’s Hospital (GDREC2019-555H-2) and was conducted in full compliance with the ethical principles of the Declaration of Helsinki.

**Fig. 1.**
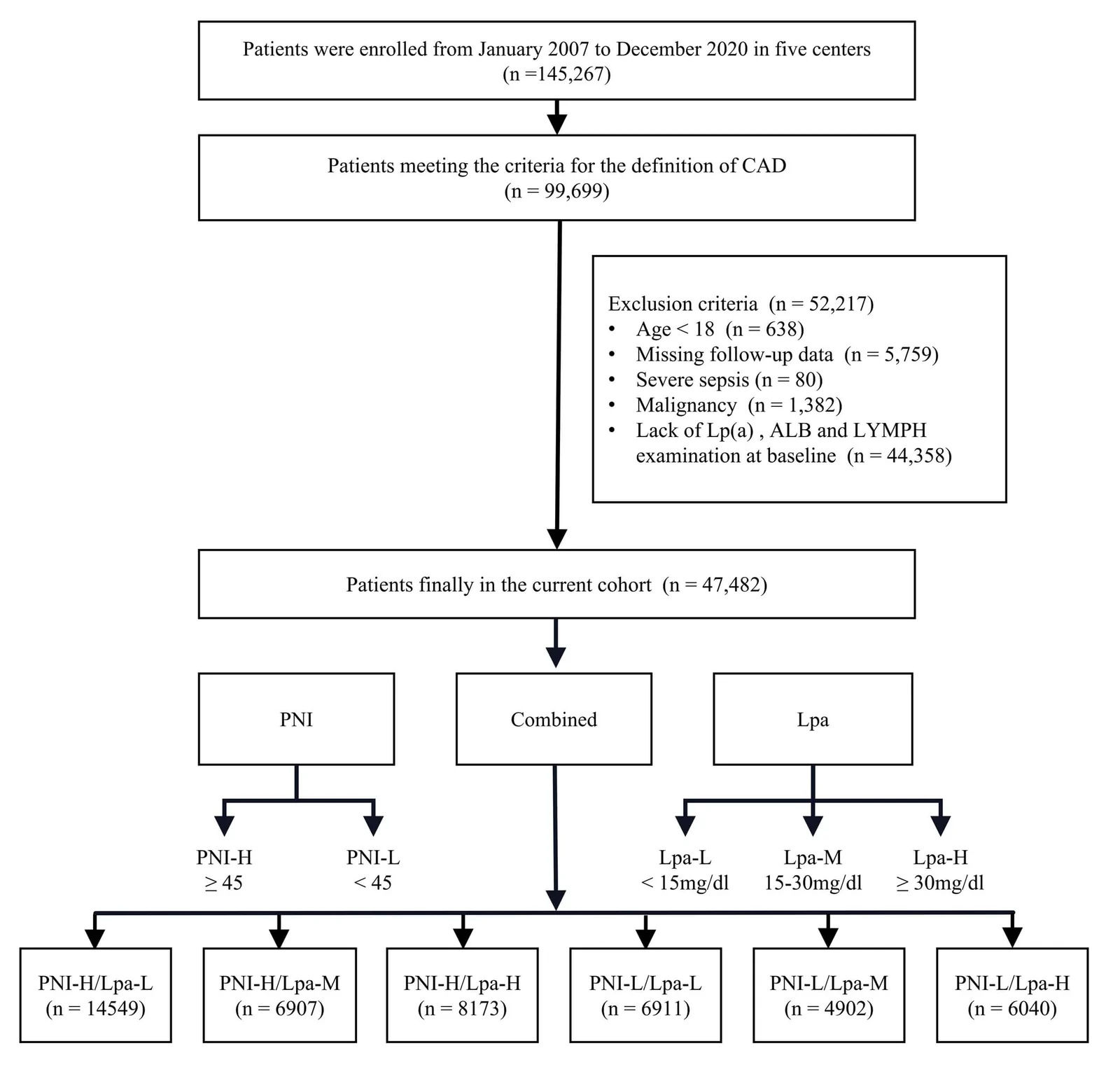
**Flow chart of the study population screening and enrollment process**. Abbreviations: CAD, coronary artery disease; Lp(a), lipoprotein(a); PNI, prognostic nutritional index; ALB, albumin; LYMPH, lymphocyte count; L, low; M, middle; H, high.

### 2.2 Data Acquisition

The primary data sources were the Electronic Clinical Management Systems at the participating institutions, from which baseline clinical data and characteristics (demographics, medical history, procedural details, laboratory findings, and discharge medications) were extracted. Laboratory parameters were acquired at the initial admission, including the key variables of serum albumin, absolute lymphocyte count, and Lp(a). Lp(a) concentrations and serum albumin were analyzed using the AU5800-3 Clinical Chemistry System (Beckman Coulter, Brea, CA, USA). Complete blood count, including absolute lymphocyte count, was performed using the XN9000-1 Hematology Analyzer (Sysmex Corporation, Kobe, Hyogo, Japan). Survival status was determined by linking the ECMS data with local official cause-specific mortality registries from the Public Security Bureau and Centers for Disease Control and Prevention, with a median follow-up of 5.2 years.

### 2.3 Exposure Definition and Grouping

PNI was used as the primary validated indicator of nutritional status, calculated with the formula: PNI = serum albumin (g/L) + 5 × absolute lymphocyte count (×10^9^/L). Lp(a) was measured as the core non-traditional lipid biomarker.

Patients were stratified into three groups based on their baseline Lp(a) level [20]: Lp(a)-Low (Lpa-L: <15 mg/dL, n = 21,460), Lp(a)-Middle (Lpa-M: ≥15 mg/dL and <30 mg/dL, n = 11,809), and Lp(a)-High (Lpa-H: ≥30 mg/dL, n = 14,213). Patients were also dichotomized according to their baseline PNI level [21]: PNI-High (PNI-H: ≥45, n = 29,629) and PNI-Low (PNI-L: <45, n = 17,853). For the assessment of joint effects, patients were further divided into 6 mutually exclusive groups based on combined Lp(a) and PNI status: Lp(a)-L/PNI-H, Lp(a)-M/PNI-H, Lp(a)-H/PNI-H, Lp(a)-L/PNI-L, Lp(a)-M/PNI-L, and Lp(a)-H/PNI-L.

### 2.4 Clinical Definitions and Outcomes

All clinical definitions followed established, standardized criteria. Chronic kidney disease (CKD) was defined as an estimated glomerular filtration rate (eGFR) ≤60 mL/min/1.73 m^2^, derived from the CKD Epidemiology Collaboration (CKD-EPI) equation [22]. Congestive heart failure (CHF) was considered present with New York Heart Association (NYHA) functional class >2 for chronic CAD and Killip class >1 in those with acute myocardial infarction (AMI) [23]. Anemia was identified by a hematocrit ≤39% in men and ≤36% in women. Dyslipidemia was determined when at least one of the following criteria was met: fasting total cholesterol ≥240 mg/dL, LDL-C >160 mg/dL, triglycerides ≥150 mg/dL, or high-density lipoprotein cholesterol (HDL-C) <40 mg/dL. AMI, diabetes mellitus, and hypertension were classified using the International Classification of Diseases, ICD-10 codes. Coronary angiographic characteristics were classified as single-vessel CAD, multivessel CAD, or left main CAD, defined as ≥50% stenosis in one, two or more, or the left main major epicardial coronary artery, respectively.

Primary endpoints included all-cause and cardiovascular mortality. All-cause mortality was determined through official death registries, while cardiovascular mortality was specifically identified from primary and secondary causes of death classified under ICD-10 codes I00–I99.

### 2.5 Statistical Analysis

Continuous variables were first tested for normality using the Shapiro-Wilk test. Normally distributed variables were expressed as the means ± standard deviation (SD) and compared using Student’s *t*-test or one-way ANOVA, as appropriate. Non-normally distributed variables were expressed as the medians (interquartile ranges [IQR]) and compared using the Wilcoxon rank-sum test or Kruskal-Wallis test. Categorical variables were shown as counts (proportions), with group differences evaluated by chi-square test or Fisher’s exact test.

Kaplan-Meier survival curves were constructed to visualize cumulative survival probabilities, and group differences were compared using the log-rank test. Univariable and multivariable Cox proportional hazards models examined associations between baseline PNI and Lp(a) levels and the risks of all-cause and cardiovascular mortality, including both the independent prognostic effects of individual markers and the joint effects of combined Lp(a)-PNI status, with low Lp(a)/high PNI used as the reference. Multivariable models were adjusted for traditional cardiovascular disease risk factors (age, sex, hypertension, diabetes, dyslipidaemia, CKD, AMI, and CHF), coronary angiographic characteristics, and the neutrophil-to-lymphocyte ratio (NLR, calculated as neutrophil count / lymphocyte count) [24,25,26,27].

To further quantitatively evaluate the incremental predictive value, time-dependent receiver operating characteristic (ROC) analyses were performed, with both variables modeled continuously. The area under the curve (AUC) was computed to compare the discriminatory power of Lp(a), PNI, and their combination. Bootstrap internal validation with 1000 resamples was applied to validate model stability and predictive performance. Model calibration was evaluated by comparing predicted risks with observed outcomes, and clinical utility was evaluated using decision curve analysis (DCA).

Potential additive interactions between PNI and Lp(a) on mortality risks were investigated using additive scale models to compute the relative excess risk due to interaction (RERI), attributable proportion due to interaction (AP), and the synergy index (SI), with 95% confidence intervals (CIs) derived via the delta method. An additive interaction was defined as RERI >0, AP >0, and SI >1, indicating that the combined effect surpassed the sum of individual effects. Subgroup analyses were performed according to age (≤65 vs >65 years), sex (male vs female), LDL-C concentration (<1.8 vs ≥1.8 mmol/L) and AMI status (no vs yes). Multiplicative interaction terms were included in multivariable Cox models to test for effect modification. Sensitivity analyses were conducted to prove the robustness of the primary findings: (1) redefining participants according to the median cutoff value of PNI for Cox regression and additive interaction analyses; (2) excluding patients with AMI and those with markedly elevated inflammatory status (high-sensitivity C-reactive protein (hs-CRP) >3.0 mg/L) to reassess the prognostic associations and interaction effects.

Two-tailed *p*-values < 0.05 were considered statistically significant. All statistical analyses were conducted using R software version 4.4.2 (R Foundation for Statistical Computing, Vienna, Austria).

## 3. Results

### 3.1 Study Population and Baseline Characteristics

This study included 47,482 participants, 29,629 (7117 women; 62.4%) were classified into the high PNI group (PNI ≥45) and 17,853 (4215 women; 37.6%) into the low PNI group (PNI <45). Table 1 summarizes the baseline demographics, clinical characteristics, biochemical parameters, and discharge medications for the overall cohort and PNI subgroups, with additional stratification by Lp(a) levels (<15 mg/dL, 15 to <30 mg/dL, ≥30 mg/dL).

**Table 1.** **Baseline clinical characteristics of the study population stratified by lipoprotein(a) and prognostic nutritional index levels**.

Characteristic		Overall	PNI ≥45				PNI <45				*p*
			All	Lp(a) <15 mg/dL	Lp(a) 15–30 mg/dL	Lp(a) ≥30 mg/dL	All	Lp(a) <15 mg/dL	Lp(a) 15–30 mg/dL	Lp(a) >30 mg/dL	
		(n = 47,482)	(n = 29,629)	(n = 14,549)	(n = 6907)	(n = 8173)	(n = 17,853)	(n = 6911)	(n = 4902)	(n = 6040)	
Demographic characteristics											
Age, years		62.8 (10.7)	60.70 (10.41)	60.73 (10.46)	61.03 (10.25)	60.35 (10.43)	66.24 (10.19)	66.31 (10.26)	66.59 (10.21)	65.86 (10.09)	<0.001
Female, n (%)		11,332 (23.9)	7117 (24.0)	3345 (23.0)	1621 (23.5)	2151 (26.3)	4215 (23.6)	1603 (23.2)	1102 (22.5)	1510 (25.0)	<0.001
Current smoker, n (%)		12,996 (27.4)	8002 (27.0)	3871 (26.6)	1882 (27.2)	2249 (27.6)	4994 (28.0)	1911 (27.6)	1381 (28.2)	1702 (28.2)	0.519
Coexisting conditions											
AMI, n (%)		9670 (20.4)	4350 (14.7)	1834 (12.6)	1172 (17.0)	1344 (16.4)	5320 (29.8)	1834 (26.5)	1549 (31.6)	1937 (32.1)	<0.001
CHF, n (%)		7363 (15.5)	3227 (10.9)	1381 (9.5)	873 (12.6)	973 (11.9)	4136 (23.2)	1428 (20.7)	1172 (23.9)	1536 (25.4)	<0.001
Hypertension, n (%)		26,514 (55.8)	16,254 (54.9)	8136 (55.9)	3761 (54.5)	4357 (53.3)	10,260 (57.5)	4018 (58.1)	2779 (56.7)	3463 (57.3)	<0.001
Diabetes mellitus, n (%)		17,406 (36.7)	10,829 (36.5)	5562 (38.2)	2441 (35.3)	2826 (34.6)	6577 (36.8)	2572 (37.2)	1771 (36.1)	2234 (37.0)	<0.001
CKD, n (%)		37,594 (79.3)	4206 (14.2)	1959 (13.5)	1010 (14.7)	1237 (15.2)	5584 (31.3)	1933 (28.0)	1565 (32.0)	2086 (34.6)	<0.001
Anaemia, n (%)		14,956 (31.5)	6137 (20.7)	2882 (19.8)	1392 (20.2)	1863 (22.8)	8819 (49.4)	3188 (46.1)	2456 (50.1)	3175 (52.6)	<0.001
Atrial fibrillation, n (%)		1887 (4.0)	946 (3.2)	516 (3.5)	225 (3.3)	205 (2.5)	941 (5.3)	383 (5.5)	266 (5.4)	292 (4.8)	<0.001
Stroke, n (%)		2801 (5.9)	1494 (5.0)	716 (4.9)	321 (4.6)	457 (5.6)	1307 (7.3)	473 (6.8)	350 (7.1)	484 (8.0)	<0.001
NYHA class											0.011
	I	4441 (17.1)	3200 (20.4)	1562 (20.7)	780 (21.1)	858 (19.4)	1241 (12.1)	493 (12.5)	345 (12.3)	403 (11.4)	
	II	16,825 (64.9)	10,382 (66.2)	5094 (67.4)	2340 (63.3)	2948 (66.7)	6443 (62.8)	2526 (64.3)	1739 (62.2)	2178 (61.7)	
	III	3905 (15.1)	1881 (12.0)	805 (10.6)	533 (14.4)	543 (12.3)	2024 (19.7)	729 (18.5)	550 (19.7)	745 (21.1)	
	IV	767 (3.0)	219 (1.4)	100 (1.3)	46 (1.2)	73 (1.7)	548 (5.3)	182 (4.6)	163 (5.8)	203 (5.8)	
Laboratory examination											
PNI		46.62 ± 5.82	50.12 ± 3.79	50.36 ± 3.90	49.95 ± 3.68	49.85 ± 3.64	40.82 ± 3.52	41.24 ± 3.24	40.71 ± 3.48	40.42 ± 3.79	<0.0001
Lp(a), mg/dL		16.99 [8.84, 35.60]	15.34 [8.10, 32.94]	8.00 [5.40, 10.97]	20.69 [17.53, 24.60]	54.30 [39.30, 81.10]	19.76 [10.40, 39.41]	8.80 [6.00, 11.66]	21.02 [17.70, 24.80]	54.30 [39.00, 82.92]	<0.001
Lymphocyte, 10^9^/L		1.91± 0.69	2.20 ± 0.66	2.21 ± 0.67	2.18 ± 0.65	2.18 ± 0.65	1.45 ± 0.46	1.44 ± 0.45	1.44 ± 0.46	1.46 ± 0.47	<0.001
Albumin, g/L		37.05 ± 4.14	39.14 ± 3.03	39.31 ± 3.01	39.04 ± 3.03	38.92 ± 3.03	33.58 ± 3.35	34.03 ± 3.15	33.49 ± 3.23	33.14 ± 3.59	<0.001
HbA1c, %		6.51 ± 1.42	6.48 ± 1.37	6.51 ± 1.39	6.46 ± 1.36	6.45 ± 1.35	6.56 ± 1.50	6.54 ± 1.48	6.54 ± 1.48	6.60 ± 1.54	<0.001
Hs-CRP		2.63 [0.90, 7.77]	1.93 [0.74, 4.87]	1.67 [0.68, 4.21]	2.14 [0.81, 5.65]	2.30 [0.84, 5.79]	4.94 [1.49, 16.80]	3.71 [1.15, 11.75]	5.72 [1.64, 21.00]	6.54 [1.96, 22.70]	<0.001
NT-proBNP		225.70 [65.14, 920.90]	132.20 [47.71, 470.90]	114.20 [42.75, 392.12]	155.60 [52.20, 565.38]	160.00 [54.57, 547.50]	657.50 [161.07, 2265.25]	452.40 [122.60, 1738.00]	782.61 [180.65, 2565.50]	857.80 [225.85, 2751.50]	<0.001
Cardiac troponin T		13.80 [7.60, 54.70]	11.23 [6.92, 26.63]	10.60 [6.77, 22.13]	11.87 [7.02, 34.75]	12.18 [7.15, 32.77]	28.30 [10.60, 323.42]	20.50 [9.36, 182.35]	30.85 [10.96, 426.75]	38.50 [12.50, 406.20]	<0.001
White blood cells, 10^9^/L		7.42 [6.17, 9.03]	7.54 [6.37, 9.01]	7.45 [6.31, 8.89]	7.58 [6.38, 9.10]	7.67 [6.45, 9.10]	7.19 [5.83, 9.08]	7.04 [5.69, 8.90]	7.25 [5.89, 9.16]	7.31 [5.92, 9.16]	<0.001
Neutrophil, 10^9^/L		4.53 [3.55, 5.91]	4.42 [3.53, 5.60]	4.34 [3.47, 5.50]	4.49 [3.56, 5.71]	4.50 [3.59, 5.70]	4.76 [3.61, 6.57]	4.62 [3.50, 6.45]	4.82 [3.65, 6.67]	4.87 [3.69, 6.59]	<0.001
Monocyte, 10^9^/L		0.59 [0.46, 0.75]	0.59 [0.47, 0.74]	0.58 [0.47, 0.73]	0.59 [0.47, 0.75]	0.59 [0.47, 0.75]	0.59 [0.46, 0.77]	0.58 [0.44, 0.75]	0.60 [0.46, 0.77]	0.60 [0.46, 0.79]	<0.001
NLR		2.43 [1.77, 3.52]	2.07 [1.59, 2.78]	2.03 [1.55, 2.71]	2.13 [1.62, 2.85]	2.12 [1.63, 2.84]	3.34 [2.38, 5.00]	3.27 [2.33, 4.91]	3.37 [2.42, 5.07]	3.40 [2.42, 5.04]	0.002
PLR		119.67 [92.22, 159.24]	106.29 [84.37, 135.00]	102.86 [81.82, 131.10]	108.61 [86.03, 137.85]	110.16 [87.74, 138.76]	151.39 [116.29, 200.83]	145.30 [112.45, 190.65]	151.97 [116.22, 201.96]	158.26 [121.19, 210.34]	<0.001
SII		539.46 [368.74, 827.69]	467.81 [332.82, 71.44]	445.47 [319.53, 636.22]	488.18 [345.17, 699.15]	493.54 [351.11, 705.21]	722.78 [472.51, 1163.69]	681.46 [444.79, 1101.79]	727.19 [487.52, 1175.10]	773.83 [506.52, 1235.28]	<0.001
Total cholesterol, mmol/L		4.55 ± 1.26	4.67 ± 1.30	4.50 ± 1.27	4.78 ± 1.28	4.89 ± 1.31	4.35 ± 1.17	4.16 ± 1.14	4.36 ± 1.12	4.57 ± 1.22	<0.001
HDL-C, mmol/L		1.01 ± 0.26	1.02 ± 0.25	1.00 ± 0.25	1.03 ± 0.25	1.04 ± 0.26	0.98 ± 0.27	0.98 ± 0.27	0.98 ± 0.27	0.99 ± 0.28	<0.001
LDL-C, mmol/L		2.86 ± 0.99	2.95 ± 1.02	2.78 ± 0.95	3.06 ± 1.04	3.15 ± 1.05	2.72 ± 0.94	2.52 ± 0.87	2.74 ± 0.90	2.92 ± 0.99	<0.001
Triglyceride, mmol/L		1.69 ± 1.27	1.84 ± 1.36	1.98 ± 1.62	1.70 ± 1.06	1.70 ± 1.02	1.45 ± 1.04	1.54 ± 1.29	1.39 ± 0.92	1.39 ± 0.76	<0.001
Hemoglobin, g/L		134.10 ± 17.46	138.45 ± 15.34	138.96 ± 15.16	138.65 ± 15.49	137.36 ± 15.48	126.89 ± 18.35	128.61 ± 17.80	126.59 ± 18.19	125.15 ± 18.91	<0.001
Apolipoprotein A, g/L		1.13 ± 0.26	1.16 ± 0.25	1.16 ± 0.25	1.17 ± 0.25	1.16 ± 0.26	1.07 ± 0.26	1.08 ± 0.26	1.06 ± 0.26	1.06 ± 0.27	<0.001
Apolipoprotein B, g/L		0.87 ± 0.25	0.89 ± 0.26	0.85 ± 0.25	0.92 ± 0.27	0.94 ± 0.27	0.83 ± 0.23	0.78 ± 0.22	0.84 ± 0.23	0.89 ± 0.24	<0.001
eGFR, mL/min/1.73 m^2^		78.2 ± 26.6	82.39 ± 24.93	83.08 ± 24.81	81.84 ± 24.85	81.61 ± 25.18	71.15 ± 27.78	72.82 ± 26.07	70.80 ± 27.87	69.52 ± 29.45	<0.001
Echocardiographic and angiographic data											
LVEF, %		59.1 ± 11.8	60.90 ± 10.69	61.39 ± 10.33	60.76 ± 10.71	60.18 ± 11.22	56.17 ± 12.99	57.47 ± 12.70	55.75 ± 13.00	55.04 ± 13.18	<0.001
Coronary lesion characteristics											
	Single-vessel, n (%)	29,358 (61.8)	18,364 (62.0)	9305 (64.0)	4346 (62.9)	4713 (57.7)	10,994 (61.6)	4366 (63.2)	3129 (63.8)	3499 (57.9)	<0.001
	Multivessel, n (%)	14,159 (29.8)	9026 (30.5)	4204 (28.9)	2043 (29.6)	2779 (34.0)	5133 (28.8)	1912 (27.7)	1323 (27.0)	1898 (31.4)	
LMCA, n (%)		3965 (8.4)	2239 (7.6)	1040 (7.1)	518 (7.5)	681 (8.3)	1726 (9.7)	633 (9.2)	450 (9.2)	643 (10.6)	
PCI, n (%)		35,300 (74.3)	21,609 (72.9)	10,295 (70.8)	5086 (73.6)	6228 (76.2)	13,691 (76.7)	5087 (73.6)	3808 (77.7)	4796 (79.4)	<0.001
CABG, n (%)		82 (0.2)	50 (0.2)	27 (0.2)	12 (0.2)	11 (0.1)	32 (0.2)	9 (0.1)	12 (0.2)	11 (0.2)	0.349
Medicine											
RASi, n (%)		33,236 (70.8)	20,882 (71.0)	10,208 (70.7)	4905 (71.6)	5769 (71.2)	12,354 (70.5)	4893 (71.9)	3353 (69.9)	4108 (69.4)	0.010
β-blocker, n (%)		37,828 (80.6)	23,972 (81.5)	11,693 (80.9)	5592 (81.6)	6687 (82.5)	13,856 (79.1)	5394 (79.3)	3764 (78.5)	4698 (79.3)	<0.001
Statins, n (%)		44,886 (94.53)	28,235 (96.0)	13,853 (95.9)	6564 (95.8)	7818 (96.4)	16,651 (95.1)	6459 (95.0)	4553 (95.0)	5639 (95.2)	<0.001
PCSK9 inhibitors n (%)		68 (0.1)	48 (0.2)	29 (0.2)	12 (0.2)	7 (0.1)	20 (0.1)	10 (0.1)	4 (0.1)	6 (0.1)	0.186
OAD, n (%)		8850 (18.9)	5774 (19.6)	3052 (21.1)	1272 (18.6)	1450 (17.9)	3076 (17.6)	1260 (18.5)	807 (16.8)	1009 (17.0)	0.027
Aspirin, n (%)		43,174 (90.9)	27,221 (92.6)	13,288 (92.0)	6351 (92.7)	7582 (93.5)	15,953 (91.1)	6159 (90.6)	4370 (91.1)	5424 (91.6)	<0.001

Abbreviations: Lp(a), lipoprotein(a); PNI, prognostic nutritional index; AMI, acute myocardial infarction; CHF, congestive heart failure; CKD, chronic kidney disease; NYHA, New York Heart Association functional classification; HbA1c, glycated hemoglobin A1c; hs-CRP, high-sensitivity C-reactive protein; NLR, neutrophil-to-lymphocyte ratio; PLR, platelet to lymphocyte ratio; SII, systemic immune-inflammation index; HDL-C, high-density lipoprotein cholesterol; LMCA, left main coronary artery; eGFR, Estimated Glomerular Filtration Rate; PCI, percutaneous coronary intervention; LDL-C, low-density lipoprotein cholesterol; LVEF, left ventricular ejection fraction; RASi, renin angiotensin system inhibitor; OAD, oral anti-diabetic drugs; NT-proBNP, N-terminal pro-B-type natriuretic peptide; CABG, coronary artery bypass grafting; PCSK9, proprotein convertase subtilisin/kexin type 9. Data are presented as the mean value (standard deviation), median [interquartile range] or number of participants (percentage).

Compared with patients in the high PNI group, those with low PNI were older, had a lower female proportion, and exhibited higher levels of hs-CRP, NT-proBNP, cardiac troponin T, neutrophil counts and Lp(a), as well as lower levels of eGFR and left ventricular ejection fraction (LVEF). They also had a greater proportion of left main coronary artery involvement. When stratified by Lp(a) levels, patients with higher Lp(a) concentrations were more likely to have higher levels of total cholesterol, LDL-C, and apolipoprotein B (all *p* < 0.05). However, there were no statistically significant differences in the utilization rates of statins and PCSK9 inhibitors across Lp(a) strata. Detailed between-group comparisons of baseline characteristics are presented in Table 1 and **Supplementary Table 1**.

Spearman correlation analysis showed no clinically relevant collinearity between Lp(a) and PNI in the overall CAD cohort (r = 0.02, *p* < 0.001), with a correlation coefficient below the pre-specified threshold for multicollinearity (r < 0.2) (**Supplementary Fig. 1**).

### 3.2 Lp(a) and Mortality Risk

We next examined the independent prognostic associations of elevated Lp(a) and reduced PNI with all-cause mortality and cardiovascular mortality. When analyzed as a continuous variable, per 1-SD increase in Lp(a) was consistently associated with increased risks of all-cause and cardiovascular mortality. For all-cause mortality, the hazard ratio (HR) was 1.15 (95% CI 1.12–1.19) in the unadjusted model, 1.16 (95% CI 1.13–1.20) in Model 1, and 1.14 (95% CI 1.10–1.17) in Model 2. For cardiovascular mortality, the association remained significant across all models, with a fully adjusted HR (aHR) of 1.16 (95% CI 1.10–1.22) per 1-SD increase in Lp(a).

Patients were stratified into three groups according to their Lp(a) level: Lp(a)-Low (<15 mg/dL, n = 21,460), Lp(a)-Middle (15 to <30 mg/dL, n = 11,809), and Lp(a)-High (≥30 mg/dL, n = 14,213). In fully adjusted models, higher Lp(a) levels were associated with a stepwise increase in mortality risk. Compared with the Lp(a)-Low group, the Lp(a)-Middle group had a 14% higher risk of all-cause mortality (aHR 1.14, 95% CI 1.05–1.21) and a 18% higher risk of cardiovascular mortality (aHR 1.18, 95% CI 1.08–1.29), while the Lp(a)-High group had a 24% higher risk of all-cause mortality (aHR 1.24, 95% CI 1.16–1.32) and a 37% higher risk of cardiovascular mortality (aHR 1.37, 95% CI 1.27–1.48) (Table 2).

**Table 2.** **COX regression models for the association of the prognostic nutritional index and lipoprotein(a) with all-cause and cardiovascular mortality among participants**.

Events/Total			All-cause mortality						CV mortality					
			Non-adjusted		Model 1^1^		Model 2^2^		Non-adjusted		Model 1^1^		Model 2^2^	
			HR (95% CI)	*p*	HR (95% CI)	*p*	HR (95% CI)	*p*	HR (95% CI)	*p*	HR (95% CI)	*p*	HR (95% CI)	*p*
Lp(a)														
	Continuous		1.15 (1.12–1.19)	<0.001	1.16 (1.13–1.20)	<0.001	1.14 (1.10–1.17)	<0.001	1.22 (1.18–1.26)	<0.001	1.23 (1.18–1.27)	<0.001	1.16 (1.10–1.22)	<0.001
	<15	1375/21460	Reference	-	Reference	-	Reference	-	Reference	-	Reference	-	Reference	-
	15–30	971/11809	1.22 (1.13–1.30)	<0.001	1.18 (1.10–1.26)	0.011	1.14 (1.05–1.21)	0.001	1.28 (1.18–1.39)	<0.001	1.24 (1.14–1.35)	<0.001	1.18 (1.08–1.29)	<0.001
	≥30	1352/14213	1.31 (1.23–1.39)	<0.001	1.33 (1.25–1.41)	<0.001	1.24 (1.16–1.32)	<0.001	1.47 (1.37–1.59)	<0.001	1.49 (1.38–1.61)	<0.001	1.37 (1.27–1.48)	<0.001
PNI														
	Continuous		0.91 (0.91–0.92)	<0.001	0.94 (0.93–0.94)	<0.001	0.95 (0.94–0.95)	<0.001	0.91 (0.90–0.92)	<0.001	0.92 (0.92–0.93)	<0.001	0.92 (0.92–0.93)	<0.001
	≥45	1336/29629	Reference	-	Reference	-	Reference	-	Reference	-	Reference	-	Reference	-
	<45	2362/17853	2.43 (2.30–2.57)	<0.001	1.87 (1.73–1.95)	<0.001	1.61 (1.51–1.71)	<0.001	2.67 (2.50–2.86)	<0.001	2.11 (1.93–2.26)	<0.001	1.76 (1.64–1.90)	<0.001
Combined														
	Lpa-L / PNI-H	927/14549	Reference	-	Reference	-	Reference	-	Reference	-	Reference	-	Reference	-
	Lpa-M / PNI-H	465/6907	1.06 (0.95–1.18)	0.321	1.06 (0.95–1.18)	0.322	1.04 (0.93–1.17)	0.475	1.07 (0.94–1.23)	0.307	1.07 (0.94–1.23)	0.307	1.05 (0.91–1.21)	0.486
	Lpa-H / PNI-H	605/8173	1.16 (1.04–1.28)	0.006	1.20 (1.08–1.33)	<0.001	1.16 (1.04–1.29)	0.001	1.23 (1.08–1.39)	0.001	1.26 (1.11–1.43)	0.001	1.21 (1.06–1.37)	0.004
	Lpa-L / PNI-L	1128/6911	2.29 (2.10–2.50)	<0.001	1.72 (1.58–1.88)	<0.001	1.53 (1.40–1.68)	<0.001	2.40 (2.16–2.67)	<0.001	1.88 (1.69–2.10)	<0.001	1.60 (1.43–1.79)	<0.001
	Lpa-M / PNI-L	917/4902	2.66 (2.43–2.91)	<0.001	1.99 (1.82–2.19)	<0.001	1.73 (1.57–1.90)	<0.001	2.95 (2.64–3.29)	<0.001	2.31 (2.06–2.58)	<0.001	1.91 (1.70–2.15)	<0.001
	Lpa-H / PNI-L	1187/6040	2.81 (2.58–3.06)	<0.001	2.21 (2.03–2.41)	<0.001	1.85 (1.69–2.03)	<0.001	3.39 (3.06, 3.76)	<0.001	2.76 (2.49, 3.06)	<0.001	2.20 (1.98–2.46)	<0.001

Abbreviations: CV, cardiovascular; HR, hazard ratio; Lp(a), lipoprotein(a); PNI, prognostic nutritional index; H, high; M, middle; L, low. ^1 ^Model 1 was adjusted for age, gender. ^2 ^Model 2 was adjusted for age, gender, hypertension, diabetes, dyslipidemia, chronic kidney disease, acute myocardial infarction, congestive heart failure, statin use, PCSK9 inhibitor use, coronary lesion characteristics, and neutrophil-to-lymphocyte ratio.

Restricted cubic spline analysis revealed significant nonlinear relationships between Lp(a) and both all-cause mortality (*p* for nonlinearity = 0.015) and cardiovascular mortality (*p* for nonlinearity = 0.001) (**Supplementary Fig. 2**). Kaplan–Meier curves with log-rank tests showed significantly higher cumulative mortality in patients with elevated Lp(a) levels (*p* < 0.001 for both endpoints) (Fig. 2A and 2D). Subgroup analyses revealed no significant multiplicative interaction between Lp(a) levels and age, sex, LDL-C concentration, diabetes, or AMI for all-cause mortality (all *p* for interaction > 0.05, **Supplementary Table 2**).

**Fig. 2.**
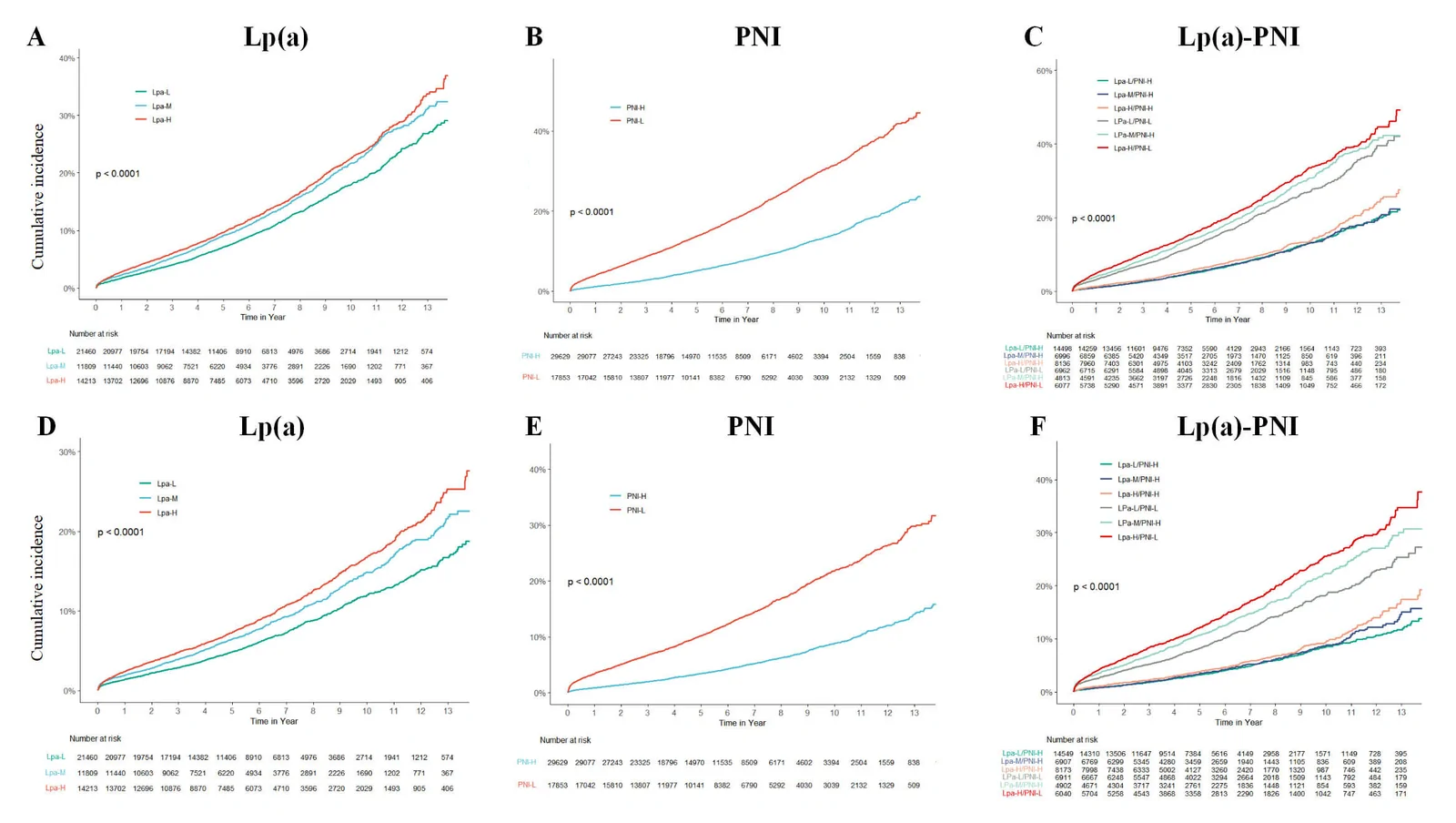
**Kaplan–Meier analysis for all-cause and cardiovascular mortality in coronary artery disease (CAD) patients, stratified by lipoprotein(a) [Lp(a)] and prognostic nutritional index (PNI)**. (A–C) The Lp(a), PNI and the Lp(a)-PNI combination for all-cause mortality risks. (D–F) The Lp(a), PNI and the Lp(a)-PNI combination for cardiovascular mortality risks. Lp(a), lipoprotein(a); H, high; L, low; PNI, prognostic nutritional index.

### 3.3 PNI and Mortality Risk

When analyzed as a continuous variable, per 1-point increase in PNI was associated with a 5% lower risk of all-cause mortality (aHR 0.95, 95% CI 0.94–0.95) and an 8% lower risk of cardiovascular mortality (aHR 0.92, 95% CI 0.92–0.93) in the fully adjusted model (both *p* < 0.001). Compared with the high PNI group, patients in the low PNI group had a 61% higher risk of all-cause mortality (aHR 1.61, 95% CI 1.51–1.71) and a 76% higher risk of cardiovascular mortality (aHR 1.76, 95% CI 1.64–1.90) after full adjustment (both *p* < 0.001) (Table 2).

Kaplan–Meier curves with log-rank tests showed significantly higher cumulative mortality in the low PNI group (*p* < 0.001 for both endpoints) (Fig. 2B and 2E). Restricted cubic spline analysis confirmed significant nonlinear associations between PNI and both mortality endpoints after full adjustment (both *p* for nonlinearity <0.001) (**Supplementary Fig. 2**). Subgroup analyses consistently validated the independent prognostic value of PNI across all predefined patient strata (**Supplementary Table 3**).

### 3.4 Joint Effects of Lp(a) and PNI on Mortality Risk

Given the significant independent prognostic value of Lp(a) and PNI, we further investigated potential biological interactions between these two biomarkers and quantitatively evaluated their additive interaction effect. Patients were divided into 6 mutually exclusive groups based on combinations of Lp(a) and PNI status, with Lp(a)-Low/PNI-High (Lpa-L/PNI-H) set as the reference group. As shown in Table 2, compared with the reference group, the isolated high Lp(a) group (Lpa-H/PNI-H) and isolated low PNI group (Lpa-L/PNI-L) both had significantly elevated risks of all-cause mortality after full adjustment in Model 2 (aHR 1.16, 95% CI 1.04–1.29, *p* = 0.001; aHR 1.53, 95% CI 1.40–1.68, *p* < 0.001, respectively). Importantly, patients with combined high Lp(a) and low PNI had the highest risk of all-cause mortality, with a crude HR of 2.81 (95% CI 2.58–3.06, *p* < 0.001) and a fully adjusted HR of 1.85 (95% CI 1.69–2.03, *p* < 0.001) in Model 2.

Kaplan–Meier curves with log-rank tests confirmed the Lp(a)-High/PNI-Low group had the highest cumulative incidence of all-cause and cardiovascular mortality. These were was significantly higher than those of all other groups (*p* < 0.001 for all comparisons) (Fig. 2C and 2F).

To further quantify the additive interaction between PNI and Lp(a), we calculated additive interaction indices (Table 3). Analysis revealed a statistically significant additive interaction for all-cause mortality, with a RERI of 0.30 (95% CI 0.14–0.43), AP of 0.12 (95% CI 0.07–0.16), and SI of 1.24 (95% CI 1.15–1.33). A stronger additive interaction was observed for cardiovascular mortality, with a RERI of 0.32 (95% CI 0.11–0.52), AP of 0.12 (95% CI 0.06–0.18), and SI of 1.24 (95% CI 1.13–1.35). The additive interaction analysis yielded consistent results across all measures, with RERI, AP, and SI each reaching statistical significance (all *p* < 0.001) for both all-cause and cardiovascular mortality.

**Table 3.** **Interactive effects of prognostic nutritional index and lipoprotein(a) on all-cause and cardiovascular mortality among participants**.

Interactive items	Interactive effect (95% CI) ^1^			
	All-cause mortality	*p*	CV mortality	*p*
RERI	0.30 (0.14–0.43)	<0.001	0.32 (0.11–0.52)	<0.001
AP	0.12 (0.07–0.16)	<0.001	0.12 (0.06–0.18)	<0.001
SI	1.24 (1.15–1.33)	<0.001	1.24 (1.13–1.35)	<0.001

Abbreviations: CV, cardiovascular; AP, proportion attributable to interaction; RERI, relative excess risk due to interaction; SI, synergy index. ^1^ All models adjusted for age, gender, hypertension, diabetes, dyslipidemia, chronic kidney disease, acute myocardial infarction, congestive heart failure, statin use, PCSK9 inhibitor use, coronary lesion characteristics, and neutrophil-to-lymphocyte ratio.

### 3.5 Predictive Performance of the Lp(a)-PNI Combination

Considering the interactive effect described above, we subsequently tested whether combining Lp(a) with PNI could further improve predictive discrimination, calibration, and clinical net benefit for long-term mortality. The discriminative performance of Lp(a) alone, PNI alone, and their combination was compared for all-cause and cardiovascular mortality using ROC curve analysis (Fig. 3). For all-cause mortality, the Lp(a)-PNI combination achieved the highest AUC of 0.726 (95% CI 0.718–0.733, *p* < 0.001), followed by PNI alone (AUC 0.681, 95% CI 0.673–0.688, *p* < 0.001), and Lp(a) alone (AUC 0.658, 95% CI 0.650–0.666, *p* < 0.001).

**Fig. 3.**
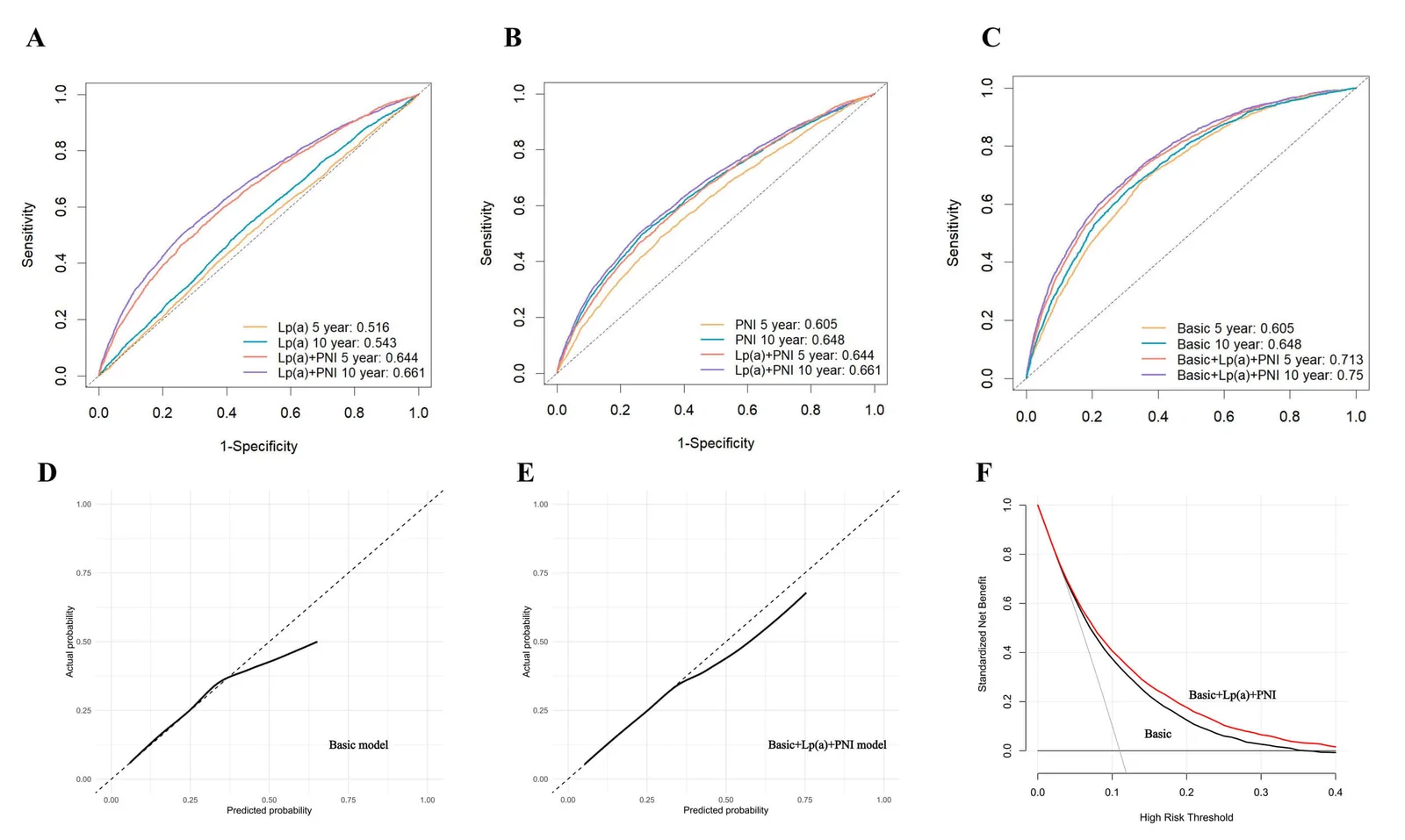
**Predictive performance of the combined lipoprotein(a) [Lp(a)] and prognostic nutritional index (PNI) for all-cause mortality in patients with coronary artery disease (CAD)**. (A) The ROC curves of the Lp(a)-PNI combination vs. Lp(a) as a marker to predict all-cause mortality risks in patients with CAD. (B) The ROC curves of the Lp(a)-PNI combination vs. PNI as a marker to predict all-cause mortality risks in patients with CAD. (C) The ROC curves of basic risk model vs. + the Lp(a)-PNI combination to predict all-cause mortality. (D) Calibration curves of basic risk model. (E) Calibration curves of basic + Lp(a) + PNI model. (F) The decision curve analysis of basic risk model vs. + the Lp(a)-PNI combination.

To evaluate the incremental predictive value, the performance of a base risk model (including all covariates in Model 2) was compared with the base model plus the Lp(a)-PNI combination for 5-year and 10-year clinical endpoints. The addition of Lp(a)-PNI combination yielded an evident improvement in model discrimination, with the time-dependent AUC rising from 0.605 to 0.713 for the 5-year endpoint and from 0.648 to 0.750 for the 10-year endpoint (all *p* < 0.001) (Fig. 3C). Calibration curves demonstrated good agreement between predicted and observed mortality risks for both models (Fig. 3D and 3E). DCA further showed that adding Lp(a) and PNI to the base model yielded a significant improvement in clinical net benefit across the full range of threshold probabilities (Fig. 3F).

### 3.6 Sensitivity Analyses

Sensitivity analyses confirmed the robustness of the primary findings (**Supplementary Table 4** and **Supplementary Table 5**). Reclassifying participants using the median PNI as the cutoff yielded similar prognostic associations and consistent additive joint effects of PNI and Lp(a) on cardiovascular and all-cause mortality. After excluding patients with AMI and those with hs-CRP >3.0 mg/L, the independent prognostic associations of Lp(a) and PNI remained consistent with the primary results, and a significant difference in mortality risk was still observed in the low PNI subgroup. However, no statistically significant additive interaction between Lp(a) and PNI was observed. These findings indicate that the core prognostic value of PNI and Lp(a) is reliable across different subpopulations, while their synergistic effect may be more pronounced in the overall cohort including patients with acute cardiac events and elevated systemic inflammation.

## 4. Discussion

Based on a large-scale, multicenter real-world cohort of patients with CAD in Southern China, this is the first study to systematically demonstrate that serum Lp(a) and PNI are not only independent prognostic markers for all-cause and cardiovascular mortality in CAD patients, but also exhibit a statistically significant additive interaction on long-term mortality risk. Specifically, CAD patients with elevated Lp(a) combined with reduced PNI had the highest risk of all-cause and cardiovascular mortality. Joint assessment of the two markers yielded a statistically significant improvement in predictive ability, although the incremental change was relatively small, and its clinical implication should be interpreted with appropriate caution.

However, the precise biological mechanisms underlying the additive interaction between dyslipidemia and nutritional status on mortality risk remain unclear. There may be several mechanism involved: Firstly, lower levels of albumin were observed in both the low PNI subgroup and high Lp(a) subgroup. Lp(a) promotes platelet activation and inhibits fibrinolysis to enhance prothrombotic potential, while hypoalbuminemia compromises the endothelial anticoagulant function. The superposition of these two risk factors may further elevate cardiovascular coagulation tendency, thereby triggering thrombosis, inducing myocardial ischemia and hypoxia, and ultimately progressing to MI [12,28,29]. Secondly, severe malnutrition is associated with increased inflammation [30]. Multiple inflammatory cytokines, including interleukin-1, interleukin-6, and tumor necrosis factor-α, exacerbate oxidative stress and vasoconstriction, leading to endothelial dysfunction and impairment of myocardial cell, thereby increasing the long-term risk of adverse outcomes in CAD patients [28,29]. Finally, there is an interaction between nutritional status and dyslipidemia. Lymphopenia directly impairs systemic immune defense and anti-inflammatory regulatory capacity, which may render the body unable to effectively counteract vascular endothelial inflammation and innate immune activation triggered by oxidized phospholipids carried by Lp(a) [31]. In turn, chronic inflammation induced by Lp(a) further accelerates systemic catabolism and increases nutritional consumption, which may further deteriorate nutritional status and lower PNI, and ultimately form a vicious cycle that may promote the progression of atherosclerosis [32].

A large number of previous studies have reported that elevated Lp(a) is an independent, causal risk factor for the onset and adverse prognosis of CAD [7,33,34,35]. Similarly, reduced PNI, a composite biomarker integrating nutritional and inflammatory status, has consistently been shown to predict adverse cardiovascular events in CAD patients [36,37,38]. However, almost all previous studies have investigated the prognostic role of each of these factors alone, while few have evaluated their joint and interactive effects on CAD outcomes. The importance of the combined assessment of lipid and nutritional biomarkers is illustrated by the “lipid paradox” phenomenon reported in a classic cohort study [39,40,41]. This phenomenon indicates that the prognostic value of lipid biomarkers cannot be interpreted independently of nutritional status, which is consistent with the findings of the present study. Furthermore, the EPIC-Norfolk study demonstrated that isolated Lp(a) has limited long-term cardiovascular predictive value, which is enhanced when combined with hs-CRP and LDL-C to identify the 20-year risk [42]. Several studies have suggested that combined assessment of Lp(a) and the cytokine interleukin-6 more accurately predicts atherosclerotic cardiovascular disease risk than either marker alone [43,44]. This indicates that the interaction between Lp(a) and inflammation is a general phenomenon that may involve the activation of the entire inflammatory pathway. It further suggests that the interaction between Lp(a) and nutritional status may be mediated by the level of systemic inflammation, supporting the key role of inflammation in this synergistic effect.

Our findings have important clinical implications for risk stratification and management of CAD patients. First, both Lp(a) and PNI retained their strong independent prognostic associations, confirming their universal predictive value across the entire hospitalized CAD population. Furthermore, the combined assessment of Lp(a) and PNI can be used for risk stratification in hospitalized CAD patients, helping to identify high-risk patients, and providing a basis for clinicians to develop individualized management strategies. However, it is critical to emphasize that this study exclusively enrolled hospitalized CAD patients, and the prognostic value of this combined assessment strategy in stable outpatient CAD populations has not been validated. This conclusion is supported by our subgroup analyses. Specifically, the statistically significant additive interaction between these two biomarkers observed in the overall cohort was attenuated and no longer detectable after excluding patients with AMI and those with hs-CRP >3.0 mg/L. These findings suggest that the synergistic effect between Lp(a) and malnutrition may be mediated by systemic inflammatory status and is significantly amplified in the context of high inflammation.

The robustness of these conclusions is supported by several strengths of our study. Based on a multicenter, large-sample real-world cohort of 47,482 CAD patients, it is one of the largest studies in this field with sufficient statistical power. Hence, the results are closer to the real-life clinical diagnosis and treatment scenario. Notably, this is the first study to systematically and quantitatively verify the additive interaction between Lp(a) and PNI on all-cause and cardiovascular mortality in CAD patients, thus filling a critical gap in previous studies. Both core biomarkers have excellent clinical accessibility. Lp(a) is a guideline-recommended cardiovascular risk marker [45], and PNI uses only two routine laboratory tests. Their low cost and simplicity ensure that our combined risk stratification strategy is highly applicable to clinical practice. Moreover, this study adopted strict multivariable confounding adjustment, restricted cubic spline nonlinear analysis, quantitative assessment of additive interaction, and sensitivity analyses, which minimized bias and ensured robust and reliable results. Importantly, the present findings offer certain potential value for clinical translation and targeted risk management. It is worth noting that existing clinical evidence has identified multiple pharmacotherapies and surgical interventions with significant regulatory effects on circulating Lp(a) levels, including pioglitazone, raloxifene, and bariatric surgery [46,47,48]. Combined with our finding that low PNI further increases the prognostic risk of elevated Lp(a), this provides a novel rationale for combined risk stratification and targeted intervention strategies in high-risk CAD patients.

## 5. Limitations

Nevertheless, this work has several limitations that should be acknowledged. First, this retrospective observational study can only establish associations between biomarkers and prognosis, not causal relationships. Although comprehensive adjustment for major confounding factors in multivariate regression and sensitivity analyses confirmed the robustness of our results, both inherent baseline disparities between subgroups and unmeasured residual confounding could not be ruled out entirely. Additionally, the exclusion of patients with incomplete clinical data may have introduced potential selection bias. Second, although this was a multicenter hospital-based cohort, all participants were exclusively hospitalized CAD patients recruited from southern China. This geographic restriction limits nationwide representativeness, and the findings cannot be directly generalized to community residents, asymptomatic populations, other ethnic groups, or different CAD subtypes. Notably, the combined assessment of Lp(a) and PNI appears to be particularly relevant and prognostic in the setting of acute or hospitalized CAD, whereas its clinical predictive value in stable outpatient CAD populations remains uncertain and requires further validation. Third, Lp(a) and PNI were measured only once at admission, and the prognostic significance of longitudinal changes was not evaluated. It is worth noting that multiple pharmacotherapies and surgical interventions have been shown to significantly modulate circulating Lp(a) levels, including pioglitazone, raloxifene, and bariatric surgery [46,47,48]. Despite adjustment for baseline medication use in our multivariable models, we cannot rule out the possibility that initiation, discontinuation, or dose adjustment of these Lp(a)-modifying treatments during follow-up may have affected the observed prognostic relationships. Furthermore, missing data for some body composition indicators limited our ability to adjust for other potential confounding factors, even though multiple imputation was performed and sensitivity analyses confirmed that our core results remained stable. Finally, our primary endpoints were limited to all-cause and cardiovascular mortality, and excluded non-fatal adverse cardiovascular events such as MI, stroke, and heart failure hospitalization. Importantly, whether targeted nutritional interventions can effectively modify cardiovascular risk and improve long-term clinical outcomes among patients with elevated Lp(a) accompanied by low PNI remains largely unknown. This unresolved issue requires further dedicated prospective investigations and clinical trials for confirmation.

Therefore, further large-scale prospective cohort studies, randomized controlled trials, multi-population studies, longitudinal repeated-measurement analyses, and expanded endpoint assessments are warranted. These should help to validate our findings, clarify causal links, confirm the heterogeneous prognostic performance of the Lp(a)-PNI combination across different CAD clinical states, and comprehensively evaluate the prognostic value of combined Lp(a) and PNI assessment, as well as explore the potential clinical benefits of nutritional intervention strategies for high-risk patients.

## 6. Conclusions

In conclusion, serum Lp(a) and PNI are independent prognostic markers for all-cause and cardiovascular mortality in CAD patients. These two factors exhibit a statistically significant additive interaction on the long-term risk of mortality. Joint assessment of Lp(a) and PNI can improve the incremental predictive value for long-term mortality, and provides reasonable evidence for optimized risk stratification in routine clinical practice.

## Data Availability

The raw data of this study cannot be publicly shared due to patient privacy protection and institutional ethical policies of the CardioRenal Improvement II (CIN-II) multicenter cohort. Reasonable requests for the relevant study data can be addressed to the corresponding authors.

## References

[1] Moran AE, Goldman L (2024). Predicting the Future Prevalence of Cardiovascular Disease: The Good, the Bad, the Known, and the Unknown. Circulation.

[2] Tsao CW, Aday AW, Almarzooq ZI, Anderson CAM, Arora P, Avery CL (2023). Heart Disease and Stroke Statistics-2023 Update: A Report From the American Heart Association. Circulation.

[3] Crea F (2023). Residual lipidic risk beyond low-density lipoprotein cholesterol: new challenges and opportunities. European Heart Journal.

[4] Man S, Zu Y, Yang X, Deng Y, Shen D, Ma Y (2025). Prevalence of Elevated Lipoprotein(a) and its Association With Subclinical Atherosclerosis in 2.9 Million Chinese Adults. Journal of the American College of Cardiology.

[5] Tsimikas S (2017). A Test in Context: Lipoprotein(a): Diagnosis, Prognosis, Controversies, and Emerging Therapies. Journal of the American College of Cardiology.

[6] Thomas PE, Vedel-Krogh S, Kamstrup PR, Nordestgaard BG (2023). Lipoprotein(a) is linked to atherothrombosis and aortic valve stenosis independent of C-reactive protein. European Heart Journal.

[7] Nordestgaard BG, Chapman MJ, Ray K, Borén J, Andreotti F, Watts GF (2010). Lipoprotein(a) as a cardiovascular risk factor: current status. European Heart Journal.

[8] He F, Huang H, Xu W, Cui K, Ruan Y, Guo Y (2024). Prognostic impact of malnutrition in patients with coronary artery disease: a systematic review and meta-analysis. Nutrition Reviews.

[9] Wu TT, Pan Y, Zhi XY, Deng CJ, Wang S, Guo XX (2024). Association between extremely high prognostic nutritional index and all-cause mortality in patients with coronary artery disease: secondary analysis of a prospective cohort study in China. BMJ Open.

[10] La Rovere MT, Maestri R, Olmetti F, Paganini V, Riccardi G, Riccardi R (2017). Additional predictive value of nutritional status in the prognostic assessment of heart failure patients. Nutrition, Metabolism, and Cardiovascular Diseases : NMCD.

[11] Xu W, Zhang L, Yang Q, Cao Y, Rao R, Lv L (2025). Associations of prognostic nutritional index with cardiovascular all-cause mortality among CVD patients with diabetes or prediabetes: evidence from the NHANES 2005-2018. Frontiers in Immunology.

[12] Bhatia HS, Becker RC, Leibundgut G, Patel M, Lacaze P, Tonkin A (2024). Lipoprotein(a), platelet function and cardiovascular disease. Nature Reviews. Cardiology.

[13] Raposeiras Roubín S, Abu Assi E, Cespón Fernandez M, Barreiro Pardal C, Lizancos Castro A, Parada JA (2020). Prevalence and Prognostic Significance of Malnutrition in Patients With Acute Coronary Syndrome. Journal of the American College of Cardiology.

[14] Li B, Zhang S, Xu C, Huang M, Xiong Z, Hui Z (2025). Association Between the Malnutrition Status and All-Cause Mortality in Patients With Moderate and Severe Aortic Stenosis: A Prospective Cohort Study. Journal of the American Heart Association.

[15] Capurso C, Lo Buglio A, Bellanti F, Vendemiale G (2024). Prognostic Nutritional Index and Instant Nutritional Assessement Are Associated with Clinical Outcomes in a Geriatric Cohort of Acutely Inpatients. Nutrients.

[16] Wen B, Njunge JM, Bourdon C, Gonzales GB, Gichuki BM, Lee D (2022). Systemic inflammation and metabolic disturbances underlie inpatient mortality among ill children with severe malnutrition. Science Advances.

[17] Reyes-Soffer G, Westerterp M (2021). Beyond Lipoprotein(a) plasma measurements: Lipoprotein(a) and inflammation. Pharmacological Research.

[18] Jneid H, Anderson JL, Wright RS, Adams CD, Bridges CR, Casey DE (2012). 2012 ACCF/AHA focused update of the guideline for the management of patients with unstable angina/non-ST-elevation myocardial infarction (updating the 2007 guideline and replacing the 2011 focused update): a report of the American College of Cardiology Foundation/American Heart Association Task Force on Practice Guidelines. Journal of the American College of Cardiology.

[19] Levine GN, Bates ER, Blankenship JC, Bailey SR, Bittl JA, Cercek B (2016). 2015 ACC/AHA/SCAI Focused Update on Primary Percutaneous Coronary Intervention for Patients With ST-Elevation Myocardial Infarction: An Update of the 2011 ACCF/AHA/SCAI Guideline for Percutaneous Coronary Intervention and the 2013 ACCF/AHA Guideline for the Management of ST-Elevation Myocardial Infarction. Journal of the American College of Cardiology.

[20] Li JJ, Ma CS, Zhao D, Yan XW, Beijing Heart Society and Expert Committee (2022). Lipoprotein(a) and Cardiovascular Disease in Chinese Population: A Beijing Heart Society Expert Scientific Statement. JACC. Asia.

[21] Onodera T, Goseki N, Kosaki G (1984). Prognostic nutritional index in gastrointestinal surgery of malnourished cancer patients. Nihon Geka Gakkai Zasshi.

[22] Delanaye P, Cavalier E, Pottel H, Stehlé T (2023). New and old GFR equations: a European perspective. Clinical Kidney Journal.

[23] Heart Failure Group of Chinese Society of Cardiology of Chinese Medical Association, Chinese Heart Failure Association of Chinese Medical Doctor Association, Editorial Board of Chinese Journal of Cardiology (2018). Chinese guidelines for the diagnosis and treatment of heart failure 2018. Zhonghua Xin Xue Guan Bing Za Zhi.

[24] Li T, Yuan D, Wang P, Zeng G, Jia S, Zhang C (2023). Association of prognostic nutritional index level and diabetes status with the prognosis of coronary artery disease: a cohort study. Diabetology & Metabolic Syndrome.

[25] Sun S, Wang Y, Pang S, Wu X (2024). Combination of the glycated hemoglobin levels and prognostic nutritional index as a prognostic marker in patients with acute coronary syndrome and type 2 diabetes mellitus. Lipids in Health and Disease.

[26] Rikhi R, Bhatia HS, Schaich CL, Ashburn N, Tsai MY, Michos ED (2023). Association of Lp(a) (Lipoprotein[a]) and Hypertension in Primary Prevention of Cardiovascular Disease: The MESA. Hypertension.

[27] Morsy MI, Friday JM, Pell JP, Lewsey J, Mackay DF, Dundas R (2026). Neutrophil-to-Lymphocyte Ratio and Mortality in Cardiovascular Disease or Cancer: A Population-Based Cohort Study. JACC. Advances.

[28] Soeters PB, Wolfe RR, Shenkin A (2019). Hypoalbuminemia: Pathogenesis and Clinical Significance. JPEN. Journal of Parenteral and Enteral Nutrition.

[29] Bevers LM, van Faassen EE, Vuong TD, Ni Z, Boer P, Koomans HA (2006). Low albumin levels increase endothelial NO production and decrease vascular NO sensitivity. Nephrology, Dialysis, Transplantation : Official Publication of the European Dialysis and Transplant Association - European Renal Association.

[30] Kalantar-Zadeh K, Stenvinkel P, Bross R, Khawar OS, Rammohan M, Colman S (2005). Kidney insufficiency and nutrient-based modulation of inflammation. Current Opinion in Clinical Nutrition and Metabolic Care.

[31] Fock RA, Blatt SL, Beutler B, Pereira J, Tsujita M, de Barros FEV (2010). Study of lymphocyte subpopulations in bone marrow in a model of protein-energy malnutrition. Nutrition (Burbank, Los Angeles County, Calif.).

[32] Lampsas S, Xenou M, Oikonomou E, Pantelidis P, Lysandrou A, Sarantos S (2023). Lipoprotein(a) in Atherosclerotic Diseases: From Pathophysiology to Diagnosis and Treatment. Molecules (Basel, Switzerland).

[33] Gilliland TC, Liu Y, Mohebi R, Miksenas H, Haidermota S, Wong M (2023). Lipoprotein(a), Oxidized Phospholipids, and Coronary Artery Disease Severity and Outcomes. Journal of the American College of Cardiology.

[34] O'Toole T, Shah NP, Giamberardino SN, Kwee LC, Voora D, McGarrah RW (2024). Association Between Lipoprotein(a) and Obstructive Coronary Artery Disease and High-Risk Plaque: Insights From the PROMISE Trial. The American Journal of Cardiology.

[35] Arsenault BJ, Kamstrup PR (2022). Lipoprotein(a) and cardiovascular and valvular diseases: A genetic epidemiological perspective. Atherosclerosis.

[36] Liu TD, Zheng YY, Tang JN, Wang W, Dai XY, Zhang JC (2022). Prognostic Nutritional Index as a Novel Predictor of Long-Term Prognosis in Patients with Coronary Artery Disease After Percutaneous Coronary Intervention. Clinical and Applied Thrombosis/hemostasis : Official Journal of the International Academy of Clinical and Applied Thrombosis/Hemostasis.

[37] Wada H, Dohi T, Miyauchi K, Jun S, Endo H, Doi S (2018). Relationship between the prognostic nutritional index and long-term clinical outcomes in patients with stable coronary artery disease. Journal of Cardiology.

[38] Kang SH, Song HN, Moon JY, Kim SH, Sung JH, Kim IJ (2022). Prevalence and prognostic significance of malnutrition in patients with acute coronary syndrome treated with percutaneous coronary intervention. Medicine.

[39] Liu Y, Liu F, Zhang L, Li J, Kang W, Cao M (2021). Association between low density lipoprotein cholesterol and all-cause mortality: results from the NHANES 1999-2014. Scientific Reports.

[40] Rong S, Li B, Chen L, Sun Y, Du Y, Liu B (2022). Association of Low-Density Lipoprotein Cholesterol Levels with More than 20-Year Risk of Cardiovascular and All-Cause Mortality in the General Population. Journal of the American Heart Association.

[41] Johannesen CDL, Langsted A, Mortensen MB, Nordestgaard BG (2020). Association between low density lipoprotein and all cause and cause specific mortality in Denmark: prospective cohort study. BMJ (Clinical Research Ed.).

[42] Kraaijenhof JM, Nurmohamed NS, Nordestgaard AT, Reeskamp LF, Stroes ESG, Hovingh GK (2025). Low-density lipoprotein cholesterol, C-reactive protein, and lipoprotein(a) universal one-time screening in primary prevention: the EPIC-Norfolk study. European Heart Journal.

[43] Bhatia HS, McParland J, Rikhi R, Tsai M, Szklo M, Tsimikas S (2025). Association of Lipoprotein(a) and Interleukin-6 With Cardiovascular Risk: MESA and UK Biobank. Journal of the American College of Cardiology.

[44] Mohammadnia N, van Broekhoven A, Bax WA, Eikelboom JW, Mosterd A, Fiolet ATL (2025). Interleukin-6 modifies Lipoprotein(a) and oxidized phospholipids associated cardiovascular disease risk in a secondary prevention cohort. Atherosclerosis.

[45] Mach F, Koskinas KC, Roeters van Lennep JE, Tokgözoğlu L, Badimon L, Baigent C (2025). 2025 Focused Update of the 2019 ESC/EAS Guidelines for the management of dyslipidaemias. European Heart Journal.

[46] Jamialahmadi T, Mirhadi E, Reiner Ž, Abass KS, Almahmeed W, S Virani S (2025). Effects of Pioglitazone On Lipoprotein(a): A Meta-analysis. Current Atherosclerosis Reports.

[47] Ferretti G, Bacchetti T, Simental-Mendía LE, Reiner Ž, Banach M, Sahebkar A (2017). Raloxifene Lowers Plasma Lipoprotein(a) Concentrations: a Systematic Review and Meta-analysis of Randomized Placebo-Controlled Trials. Cardiovascular Drugs and Therapy.

[48] Jamialahmadi T, Reiner Ž, Alidadi M, Kroh M, Almahmeed W, Ruscica M (2022). The Effect of Bariatric Surgery on Circulating Levels of Lipoprotein (a): A Meta-analysis. BioMed Research International.

