# Supplementary material for: Lipoprotein(a) and Prognostic Nutritional Index Levels Exhibit Interactive and Joint Effects on Mortality Risk in Patients With Coronary Artery Disease: Findings From a Real-World Study
Source: Rev Cardiovasc Med. 2026 Oct 16;27(10):51948. doi: 10.31083/RCM51948 (PMC13732664; doi:10.31083/RCM51948)
Supplement: Supplementary file 1 [file 2153-8174-27-10-51948-s1.zip › Supplementary Material.docx]

**Supplementary Materials**

**Supplementary Table 1** Baseline characteristics stratified by Lp(a) and PNI level.

**Supplementary Table 2 & 3** Subgroup analysis of association between the Lp(a) and PNI and the risk of all-cause mortality.

**Supplementary Fig. 1** Correlations between Lp(a), total cholesterol, CONUT ,PNI, its components and LDL-C.

**Supplementary Fig. 2** Nonlinear associations of prognostic nutritional index and lipoprotein(a) with all-cause and cardiovascular mortality.

**Supplementary Table 1**. Baseline characteristics between survivors and non-survivors in patients with coronary artery disease.

| **Characteristic** | **Overall** | **Survivors** | **Non-Survivors** | ***P*-value** |
| --- | --- | --- | --- | --- |
|  | **(N=**47,482) | **(N=42,253)** | **(N=5229)** |  |
| **Demographic characteristics** |  |  |  |  |
| Age, years | 62.8 (10.7) | 62.08 (10.53) | 68.46 (10.05) | <0.001 |
| Female, n (%) | 11332 (23.9) | 10220 (24.2) | 1112 (21.3) | <0.001 |
| Current smoker, n (%) | 12996 (36.2) | 11370 (35.9) | 1626 (38.0) | 0.006 |
| **Coexisting conditions** |  |  |  |  |
| AMI, n (%) | 9670 (20.4) | 8519 (20.2) | 1151 (22.0) | 0.002 |
| PCI, n (%) | 35300 (74.3) | 31514 (74.6) | 3786 (72.4) | <0.001 |
| CHF, n (%) | 7363 (15.5) | 5969 (14.1) | 1394 (26.7) | <0.001 |
| Hypertension, n (%) | 26514 (55.8) | 23301 (55.1) | 3213 (61.4) | 0.002 |
| Diabetes mellitus, n (%) | 17406 (36.7) | 14986 (35.5) | 2420 (46.3) | <0.001 |
| CKD, n (%) | 37594 (79.3) | 7542 (17.9) | 2248 (43.1) | <0.001 |
| Anaemia, n (%) | 14956 (31.5) | 12356 (29.2) | 2600 (49.7) | <0.001 |
| Atrial fibrillation, n (%) | 1887 (4.0) | 1474 (3.5) | 413 (7.9) | <0.001 |
| Stroke, n (%) | 2801 (5.9) | 2292 (5.4) | 509 (9.7) | <0.001 |
| **Laboratory examination** |  |  |  |  |
| Lymphocyte, 10^9^/L | 46.62 ± 5.82 | 1.94 ± 0.69 | 1.74 ± 0.70 | <0.001 |
| ALB, g/L | 16.99 [8.84, 35.60] | 47.51 ± 4.98 | 34.90 ± 2.75 | <0.001 |
| PNI | 1.91± 0.69 | 47.04 ± 5.63 | 43.23 ± 6.22 | <0.001 |
| Lipoprotein(a), mg/dL | 37.05 ± 4.14 | 16.50 [8.64, 34.72] | 23.97 [12.96, 45.70] | <0.001 |
| HbA1c, % | 6.51 ± 1.42 | 6.48 ± 1.40 | 6.78 ± 1.54 | <0.001 |
| Total cholesterol, mmol/L | 4.55 ± 1.26 | 4.57 ± 1.27 | 4.42 ± 1.19 | <0.001 |
| HDL-C, mmol/L | 1.01 ± 0.26 | 1.01 ± 0.26 | 0.98 ± 0.27 | <0.001 |
| LDL-C, mmol/L | 2.86 ± 0.99 | 2.88 ± 1.00 | 2.72 ± 0.95 | <0.001 |
| Triglyceride, mmol/L | 1.69 ± 1.27 | 1.71 ± 1.28 | 1.55 ± 1.12 | <0.001 |
| Hemoglobin,g/L | 134.10 ± 17.46 | 135.07 ± 16.86 | 126.26 ± 20.04 | <0.001 |
| APOA, g/L | 1.13 ± 0.26 | 1.14 ± 0.26 | 1.06 ± 0.26 | <0.001 |
| APOB, g/L | 0.87 ± 0.25 | 0.87 ± 0.25 | 0.84 ± 0.23 | <0.001 |
| eGFR, mL/min/1.73 m^2^ | 78.2 ± 26.6 | 80.00 ± 25.86 | 63.34 ± 27.82 | <0.001 |
| LVEF,% | 59.1 ± 11.8 | 59.92 ± 11.19 | 52.76 ± 14.58 | <0.001 |
| **Medicine** |  |  |  |  |
| RASi, n (%) | 33236 (70.8) | 29689 (70.7) | 3547 (71.7) | <0.001 |
| β-blocker, n (%) | 37828 (80.6) | 33908 (80.8) | 3920 (79.2) | 0.009 |
| Statins, n (%) | 44886 (95.7) | 40215 (95.8) | 4671 (94.4) | <0.001 |
| Aspirin, n (%) | 43174 (92.0) | 38773 (92.4) | 4401 (88.9) | <0.001 |

**Abbreviations:** AMI, acute myocardial infarction; PCI, percutaneous coronary intervention; CHF, congestive heart failure; CKD, chronic kidney disease; HDL-C, high-density lipoprotein cholesterol; LDL-C, low-density lipoprotein cholesterol; RASi, renin angiotensin system inhibitor.

Data are presented as the mean value (standard deviation), median [interquartile range] or number of participants (percentage).

**Supplementary Table 2.** Subgroup analyses of association between the Lp(a) and the risk of all-cause mortality.

| **Variables** | **N (%)** | **HR (95%CI)** | ***P*** | **P for interaction** |
| --- | --- | --- | --- | --- |
|  |  |  |  |  |
| **Age ≥ 65** |  |  |  | 0.859 |
| No | 26136 (55.04) | 1.15 (1.11 ~ 1.19) | <0.001 |  |
| Yes | 21346 (44.96) | 1.17(1.11 ~ 1.23) | <0.001 |  |
| **Gender** |  |  |  | 0.879 |
| Male | 36150 (76.13) | 1.16 (1.12 ~ 1.20) | <0.001 |  |
| Female | 11332 (23.87) | 1.15(1.08 ~ 1.23) | <0.001 |  |
| **LDL-C ≥ 1.8 mmol/L** | |  |  | 0.291 |
| No | 5718 (12.04) | 1.16 (1.08 ~ 1.26) | 0.005 |  |
| Yes | 41758 (87.96) | 1.16 (1.13 ~ 1.20) | <0.001 |  |
| **AMI** |  |  |  | 0.355 |
| No | 37812 (79.64) | 1.15 (1.11 ~ 1.19) | <0.001 |  |
| Yes | 9670 (20.36) | 1.16 (1.09 ~ 1.24) | 0.009 |  |
| **DM** |  |  |  | 0.199 |
| No | 30076 (63.33) | 1.13 (1.08 ~ 1.18) | 0.002 |  |
| Yes | 17406 (36.67) | 1.21 (1.16 ~ 1.26) | 0.003 |  |

**Supplementary Table 3.** Subgroup analyses of association between the PNI and the risk of all-cause mortality.

| **Variables** | **N (%)** | **HR (95%CI)** | ***P*** | **P for interaction** |
| --- | --- | --- | --- | --- |
|  |  |  |  |  |
| **Age ≥ 65** |  |  |  | <0.001 |
| No | 26136 (55.04) | 0.94 (0.93 ~ 0.94) | <.001 |  |
| Yes | 21346 (44.96) | 0.91 (0.90 ~ 0.94) | <.001 |  |
| **Gender** |  |  |  | 0.046 |
| Male | 36150 (76.13) | 0.92 (0.91 ~ 0.92) | <.001 |  |
| Female | 11332 (23.87) | 0.90 (0.89 ~ 0.91) | <.001 |  |
| **LDL-C ≥ 1.8 mmol/L** | |  |  | 0.491 |
| No | 5718 (12.04) | 0.92 (0.91 ~ 0.92) | 0.003 |  |
| Yes | 41758 (87.96) | 0.92 (0.91 ~ 0.92) | <.001 |  |
| **AMI** |  |  |  | 0.158 |
| No | 37812 (79.64) | 0.91 (0.91 ~ 0.92) | <.001 |  |
| Yes | 9670 (20.36) | 0.92 (0.91 ~ 0.92) | <.001 |  |
| **DM** |  |  |  | 0.220 |
| No | 30076 (63.33) | 0.91 (0.91 ~ 0.92) | 0.004 |  |
| Yes | 17406 (36.67) | 0.91 (0.91 ~ 0.92) | <.001 |  |

**Supplementary Table 4.** Sensitivity analysis for prognostic nutritional index and lipoprotein(a) with all-cause and cardiovascular mortality

|  | **All-cause mortality** |  |  | **Cardiovascular mortality** | |
| --- | --- | --- | --- | --- | --- |
|  | **Adjusted**  **HR(95%CI)** |  |  | **Adjusted**  **HR(95%CI)** |  |
|  |  | ***P*** |  |  | ***P*** |
| **Dichotomous PNI^1^** | | | | | |
| Lpa-L / PNI-H | Reference | - |  | Reference | - |
| Lpa-M / PNI-H | 1.03 (0.91-1.18) | 0.630 |  | 1.01 (0.86-1.19) | 0.893 |
| Lpa-H / PNI-H | 1.17 (1.04-1.32) | 0.012 |  | 1.21 (1.04-1.41) | 0.011 |
| Lpa-L / PNI-L | 1.47 (1.34-1.62) | <0.001 |  | 1.52 (1.35-1.71) | <0.001 |
| Lpa-M / PNI-L | 1.66 (1.50-1.84) | <0.001 |  | 1.83 (1.62-2.07) | <0.001 |
| Lpa-H / PNI-L | 1.77 (1.60-1.94) | <0.001 |  | 2.06 (1.83-2.31) | <0.001 |
| **Exclude AMI and elevated hs-CRP (> 3 mg/L)** | | | | | |
| Lpa-L / PNI-H | Reference | - |  | Reference | - |
| Lpa-M / PNI-H | 0.92 (0.70-1.20) | 0.536 |  | 1.01 (0.73-1.40) | 0.970 |
| Lpa-H / PNI-H | 0.99 (0.77-1.28) | 0.939 |  | 1.00 (0.73-1.37) | 0.987 |
| Lpa-L / PNI-L | 1.59 (1.28-1.98) | <0.001 |  | 1.64 (1.25-2.15) | <0.001 |
| Lpa-M / PNI-L | 1.62 (1.24-2.12) | <0.001 |  | 1.49 (1.06-2.11) | 0.022 |
| Lpa-H / PNI-L | 1.50 (1.15-1.95) | <0.001 |  | 1.88 (1.39-2.55) | <0.001 |

**^1^**PNI grouped by the median.

Adjusted for age, gender, hypertension, diabetes mellitus, dyslipidemia, chronic kidney disease, congestive heart failure, statin use, PCSK9 inhibitor use, coronary lesion characteristics, and neutrophil-to-lymphocyte ratio.

**Supplementary Table 5.** Additive effects of prognostic nutritional index and lipoprotein(a) on all-cause mortality and cardiovascular mortality.

| **Interactive items** | **Interactive effect (95% CI) ^1^** | | | | |
| --- | --- | --- | --- | --- | --- |
|  | **All-cause mortality** | ***P*** | **CV mortality** |  | ***P*** |
| **Dichotomous PNI^1^** | | | | | |
| RERI | 0.30 (0.14 - 0.43) | <0.001 | 0.32 (0.11 - 0.52) |  | 0.002 |
| AP | 0.12 (0.07 - 0.16) | <0.001 | 0.12 (0.06 - 0.18) |  | <0.001 |
| SI | 1.24 (1.15 - 1.33) | <0.001 | 1.24 (1.13 - 1.35) |  | <0.001 |
| **Exclude AMI and elevated hs-CRP (> 3 mg/L)** | | | | | |
| RERI | 0.27 (-0.17–0.63) | 0.187 | 0.45 (-0.13–1.04) |  | 0.131 |
| AP | 0.20 (-0.14–0.33) | 0.095 | 0.22 (-0.04–0.48) |  | 0.097 |
| SI | 1.23 (0.67–2.26) | 0.572 | 1.71 (0.75–3.93) |  | 0.382 |

**Abbreviations**: CV, cardiovascular; AP, proportion attributable to interaction; RERI, relative excess risk due to interaction; SI, synergy index; PNI, prognostic nutritional index.

^1^ All models adjusted for age, gender, hypertension, diabetes, dyslipidemia, chronic kidney disease, acute myocardial infarction, congestive heart failure, statin use, PCSK9 inhibitor use, coronary lesion characteristics, and neutrophil-to-lymphocyte ratio.

**Supplementary Fig. 1** Correlations between Lp(a), total cholesterol, CONUT ,PNI, its components and LDL-C.

Lymphocyte count and albumin are components of PNI. *p-value for spearman correlation < 0.001.

**Supplementary Fig. 2**. Nonlinear associations of prognostic nutritional index and lipoprotein(a) with all-cause and cardiovascular mortality.

**A - B**. RCS analysis of the PNI and Lp(a) for all-cause mortality among patients with CAD; **C - D.** RCS analysis of the PNI and Lp(a) for cardiovascular mortality among patients with CAD; **E - F**. The ROC curves of PNI and Lp(a) as markers for predicting all-cause mortality among patients with CAD; **G - H**. The ROC curves of PNI and Lp(a) as markers for predicting CV mortality among patients with CAD.
